# Methods of effective low-level laser therapy in the treatment of patients with bronchial asthma (literature review)

**DOI:** 10.37796/2211-8039.1000

**Published:** 2020-03-28

**Authors:** Sergey Vladimirovich Moskvin, Aleksandr Agubechirovich Khadartsev

**Affiliations:** a O.K. Skobelkin State Scientific Center of Laser Medicine under the Federal Medical Biological Agency, Moscow, 121165, Russia; b Medical Institute, Tula State University, Tula, 300028, Russia

**Keywords:** bronchial asthma, low-level laser therapy

## Abstract

Bronchial asthma is an autoimmune disease, one of the most common and practically non-treatable by standard methods. At present, the used drugs only maintain a state of temporary remission, simultaneously having a negative effect on various organs and structures and causing side effects.

At the same time, the experts have ignored more than 50 years of successful experience of low-level laser therapy, the results of hundreds of studies proving the effectiveness of the method in treating patients with all forms of bronchial asthma. It is proved that therapeutic and periodic (2–4 per year) courses of low-level laser therapy can significantly decrease the frequency and severity of attacks, reduce or cancel the reception of medicines, as well as negative consequences.

In this brief review, only some part of studies is given as an example; pediatrics issues are almost not discussed. However, the review clearly demonstrates that various methods of laser illumination (specific techniques are given) make it possible to influence almost all the known pathogenesis of the disease, and low-level laser therapy is a truly effective method of treatment.

We note that there are very few publications published on the topic outside of Russia. Russian scientists, as always, are ahead of world science and low-level laser therapy practice.

Bronchial asthma (BA) is one of the most common diseases of the respiratory system in children and adults. The life-long progression, the high rate of aggravation and the often serious condition at the height of an asthma attack, the limited occupational aptitude and other characteristics increase the social significance of the disease. According to the latest epidemiological studies, more than 339 million people in the world suffer from BA [1]; 7.5% of the population in the USA is afflicted with it, of which 1.8 million people are hospitalized annually [2]. In Russia, according to epidemiological studies, asthma affects about 7 million people (5–7% of adults and 10–15% of children) [3]. Many patients have pronounced hormone dependence and/or various hormone-related disorders. Reduced levels of cortisol, testosterone, DHEA-s and estrogen in patients with asthma below the normal range are prognostic criteria for the deterioration of their quality of life [4,5].

Regardless of the severity, BA is a chronic inflammatory disease of the respiratory passages (RPs), occurring with the participation of mast cells, eosinophils and T-lymphocytes, the release of a large number of inflammatory mediators (Fig. 1). Inflammation of the RPs causes their hyperreactivity, bronchial obstruction, and respiratory symptoms. With hyperactivity RPs narrow too easily and/ or strongly in response to the influence of provoking factors. Bronchial obstruction is caused by the following mechanisms: acute bronchospasm, edema of the bronchial wall, obstruction by mucus and remodeling of the bronchial wall. Recurrent exacerbations of asthma are based on inflammation of the bronchi, their remodeling and impaired neurogenic control. An exacerbation of BA is associated with increased inflammation of the RPs and can be induced by a respiratory infection, exposure to allergens, or occupational sensitizing factors. Atopy, the production of excess IgE in response to exposure to exogenous allergens, is an important factor predisposing to the development of BA [3].

According to most experts, for successful treatment of patients with asthma, it is sufficient to properly divide patients according to the disease severity and adjust drug dosages and prescribed regimens for β2-agonists and systemic or inhaled glucocorticosteroids (GCS) that have remained for decades [2]. However, there has only been an increase in the number of patients throughout the world, and so far not a single patient has really been cured, i.e., no asthma attacks have been cured permanently. The task is only to provide emergency care [3]. The situation is complicated by the side effects that all drugs have. Even the use of inhaled corticosteroids comes with side effects, especially when consuming high dosages. The resulting systemic absorption often leads to a blockade of the hypothalamic-pituitary-adrenal system and the development of complications such as glaucoma, cataracts, osteoporosis and various skin lesions [6–11].

The development of intoxication and hypoxia, which impede the normal functioning of the immune system, is of great importance in BA pathogenesis. The effect of endotoxins on neutrophils is determined by the severity of all bronchopulmonary diseases, including bronchial asthma. The greatest changes concern molecules of average weight, circulating immune complexes (CIC), lipid peroxidation (LPO) and the toxicity index [12,13]. The increased content of endotoxins in the blood of asthma patients leads to a violation of the albumin-binding capacity and increased intoxication, as evidenced by a sharp increase in the albumin toxicity index [12]. The involvement of histamine and serotonin in BA pathogenesis is generally recognized [14]. The asthma exacerbation phase is characterized by increased histamine and serotonin levels in the blood and plasma, which is accompanied by the development of an intense allergic reaction, reduced phagocytic ability (both in phagocytic number and phagocytic index) and an increased number of NBT-positive neutrophils [12].

A ramified chain of events plays a significant role in the pathogenesis of asthma, depending on the functioning of almost all cellular elements of peripheral blood. The number of normal discocytes decreases; the abnormal shapes of erythrocytes with high cholesterol content on the membrane predominate; their deformability decreases, and their aggregation ability increases [15,16]. The percentage of abnormal shapes of erythrocytes (echinocytes, teardrop-shaped and target cells, ovalocytes and spherocytes) grows against the background of a decrease in the number of discocytes and an increase in the total number of immature reticulocytes, resulting in the impaired oxygen transport function of the blood and increased hypoxia [17].

According to our data, the main links of the mechanism of therapeutic action in comprehensive low-level laser therapy (LLLT) include the reduction of side effects in the form of local or systemic reactions, the improvement in the indicators of external respiratory function (ERF) and central hemodynamics, positive immune changes, the improvement in adrenal cortex function and the reduction in allergen-specific sensitivity and nonspecific hyperreactivity [18,19].

The therapeutic effect of low-intensity laser illumination (LILI) on BA, according to some authors, begins with the response of immunocompetent cells. The mechanism of formation of the cellular response to laser screening finds its manifestation in the initial phase of the development process, is associated with changes in the activity of enzymes and the structure of plasma membranes, and includes the following steps: molecular membrane rearrangements – reducing cholesterol and fatty acid saturation factor, increasing the proportion of phospholipids and reducing microviscosity; functional modification of membranes – changes in lipid-protein interactions and increased trans-membrane potential; and functional modification of cells – an increase in the phagocytic index and neutrophil count [12,13].

As our studies have shown, exposure to LILI in various modes allows for extremely effective increase in the deformability of erythrocyte membranes through their structural rearrangement. At the same time, more than 90% of erythrocytes with dysfunctional morphology restore their normal discoid shape, which enables reduction of the level of hypoxia [20].

The pathogenetic validity of various methods of LLLT in the comprehensive treatment of patients with asthma was confirmed by numerous studies [21–26]. Some national standards and clinical practice guidelines include almost all known methods: exposure to LILI in the projection of the thymus, adrenal glands, carotid sinus and Zakharyin-Ged zones, laser acupuncture, as well as intravenous laser blood illumination (ILBI) with red (635 nm wavelength) spectrum, and extracorporeal ultraviolet blood illumination (UVBI) [27,28].

Laser therapy should be considered as a multi-component and pathogenetically substantiated treatment of asthma patients, enabling the reversal of the main symptoms of the disease more quickly with an earlier cancellation or reduction of the drug dosage. These measures contribute to the complete recovery of electrophoregrams, rapid reduction of the elevated sialic acid, seromucoid and ceruloplasmin level and enhanced kinin-kallikrein system activity. After the LLLT course, a more pronounced positive dynamics is observed in the external respiration function (a decrease in the phenomena of bronchial obstruction). In addition to a faster onset and lengthening of remission periods, LLLT allows the body resistance to colds and meteorological factors to increase [26].

The exposure to pulsed IR LILI also has an immunomodulatory effect, along with a positive clinical effect. The latter is manifested by increased metabolic and mitotic activity of lymphocytes, changes in the expression and affinity of E-lymphocyte receptors and the serum concentrations of IgM. Neutrophil phagocytosis was significantly enhanced in the immune status of patients after the treatment. From 1989 to 1994 15,526 patients with various immunopathologies, including 2875 adults and 12,651 children, were treated in several medical clinics in Smolensk and Moscow (Russia) [29].

We do not significantly address the topic of treating asthma in children; it deserves a detailed study in a special review, since treatment approaches differ significantly. In this article we only cite several publications from different Russian scientific schools as an example [30–41].

It should be noted that in the English-language publications, and there are very few of them, only laser acupuncture is used and only in the treatment of children [42–49]. In Russia, laser acupuncture is also in active use [50,51], however, most frequently as part of the comprehensive treatment with the adjustment of techniques [52], which is more logical and effective.

A systematic review (search on Cochrane Library, Medline, EMBASE, AMED, CINAHL, CNKI, VIP electronic databases through February 2012), gives 13 randomized placebo-controlled trials (RCTs), the results of which do not prove or disprove the effectiveness of laser acupuncture for the treatment of bronchial asthma in children (the data are very contradictory) [53]. However, this is not surprising, since none of the publications describe the use of optimal parameters of laser illumination for laser acupuncture: 635 nm wavelength, power of 2–3 mW, exposure for 20–40 s per one corporal point, and the maximum permissible values are exceeded manifold, both in terms of laser power and exposure time.

Experimental English-language publications are also few in numbers [54–59], but they are extremely important for understanding the mechanisms of the biological action of LILI.

There are hundreds of times more studies and publications of Russian scholars that not only prove the highest efficiency of laser therapy, but also substantiate the optimal parameters of laser illumination techniques, the principles of drawing up optimal therapeutic schemes, based on many factors. There is nothing similar elsewhere in the world.

Many experts believe that ILBI is the most universal and effective method of laser therapy for asthma patients. The main advantage of ILBI is a significant reduction in the amount of medications taken and a decrease in the number of asthma attacks after treatment [60–62], which is closely related to the severity of the disease and the applied option of the LLLT technique [63].

Table 1 presents the treatment outcomes for three groups of patients after 10 daily laser therapy procedures for such daily average indicators (what fold decrease was shown), as the frequency of asthma attacks and the daily dose of β2-agonists. With regard to the GCS, the data is only qualitative: “significant” dose reduction and the ability to make a “soft” transition from systemic to inhaled medications [63; 64].

*Group 1.* Patients with mild and moderate-severe atopic BA and allergic rhinitis were exposed to continuous LILI (633 nm wavelength, power of 6 mW) endonasally for 5 min per each nasal passage.

Group 2. Patients with mild, moderate-severe and severe atopic and mixed BA were exposed percutaneously to pulsed IR LILI (890 nm wavelength, power of 5 W, pulse repetition frequency of 150–3000 Hz): in the thoracic area, in the areas of projection of the adrenal glands (the lumbar area at the level of Th_12_ – L_2_), of the thymus (the sternum area at the level of the second rib attachment) and the vascular bundle (the left supraclavicular area).

*Group 3.* Patients with moderate-severe and severe mixed and atopic BA were exposed to ILBI-635 (635 nm wavelength, power of 3 mW, 45 min exposure time).

Patients demonstrated positive dynamics in the course of the disease: the number of nocturnal asthma and dyspnea symptoms was reduced, the non-productive cough disappeared, and lung auscultation was normalized. The clinical efficacy of LLLT was confirmed by the ERF studies, and the sputum leukocyte and eosinophil counts also decreased in the patients [64].

Comparative evaluation of the clinical efficacy of LLLT in the comprehensive treatment of asthma patients indicates the need for a differentiated use of various methods depending on the form and severity of the disease [63]. In our opinion, it is always better to apply combined and combinative techniques.

Laser blood illumination (LBI) and most often its intravenous option (ILBI) is the most common method of laser therapy, which is used to treat BA patients. The first successful intravenous laser blood illumination with continuous LILI of the red spectrum (633 nm wavelength, ILBI-635) was performed in patients with bronchial asthma in the early 1980s, that is, immediately after the technique had appeared [65,66].

ILBI-635 is most effective in patients with the atopic variant of bronchial asthma, who show no effect from specific hyposensitization therapy. In steroid-dependent patients, ILBI makes it possible to reduce the dose of glucocorticosteroids or to discontinue them at all, increasing sensitivity to other medications. Laser therapy can be carried out in any phase of the disease and as the prophylactic treatment in BA patients who have sensitization to plant pollen (prior to the pollination period). LLLT has an immunomodulatory effect, adjusts the ratio between the oxidant and antioxidant systems, and normalizes the indicators of the respiratory function. The application of ILBI in the comprehensive treatment of BA patients can reduce the number of days of disability and lengthen remission periods by 2.4 times [66].

A hyperviscosity syndrome has been reported to occur in BA patients: increased whole blood viscosity at low shear rates, reduced deformability and suspension stability of erythrocytes, their increased ability to hyperaggregate, echinocytosis, the tendency of platelets to slow and weakly reversible aggregation [61]. Since the possibilities of exposure to LILI that normalize blood rheology are well known, S.A. Borzenkov (2000) [61] used ILBI-635 (wavelength 633 nm, power of 2 mW, 30 min exposure time, 10 procedures per a treatment course daily) in the comprehensive treatment of BA patients with positive clinical outcomes that correlated with normalized rheological parameters. The minimum shear stress decreased by 14%, whole blood viscosity reduced (at a shear rate of 1 cP - by 17%, at a shear rate of 9 cP - by 12%, at a shear rate of 25 cP -by 21%, at a shear rate of 100 cP - by 22%, and at a shear rate of 256 cP - by 28%), single-unit RBC count decreased by 47%, and the echinocyte count reduced by 49% as well, the RBC deformability increased by 1.2% and the non-aggregated RBC count increased by 1.18%.

But at the same time, no effect on platelet aggregating properties was found in BA patients. The application of ILBI in the comprehensive treatment of asthma patients also enables decrease in the drug dosage taken by more than 20% and reduction of hospital lengths of patients’ stay on average by 2.91 days.

The use of ILBI-635 (633 nm wavelength, 1–1.5 mW, 30 min exposure time, 10 procedures per a treatment course daily) to treat asthma enables to obtain a more pronounced normalizing effect on bronchial patency, reduce the degree of hypoxemia, contribute to the improvement of the clinical picture of the disease [17].

The synergistic effect of medication and laser therapies on central hemodynamics, microcirculation and blood rheological properties makes it possible to cancel prolonged β2-agonists and reduce doses of systemic GCS [67], when prescribing ILBI sessions, which is essential to compensate for the negative effect of glucocorticoid therapy on the morphofunctional state of endobronchial microhemocirculation [68].

ILBI-635 is known to have pronounced immunomodulatory properties associated with the effect of illumination on the lymphoid elements of peripheral blood, which is also indicated for BA patients. In addition, the content of physiologically active substances, including glucocorticoid hormones, is also changed in these patients. The level of total 11-oxycorticosteroids (11-OCS) increases from (2–20). 10^−5^ g/l to (10–50)·10^−5^ g/l in the patients' serum after five procedures of ILBI [69].

In patients with asthma in the acute phase, the balance between LPO and the antioxidant system (AOS) is disturbed, and the antioxidant activity of blood significantly decreases [12]. There is an oxidative stress, which is expressed in significant (exceeding the average in healthy people by 12 times) hyperproduction of free-radical metabolites against the background of 20% decreased activity of intracellular antioxidant enzymes. The use of inhaled GCS with the standard treatment of BA patients leads to the positive dynamics of clinical and functional parameters, but does not have an appreciable impact on LPO and AOC parameters. In standard therapy using systemic GCS patients show a significant decrease in LPO (by 33% of baseline values) with simultaneous inhibition of AOC parameters (by 12% of baseline values). When combining systemic and inhaled GCS, LPO parameters decrease less (by 25%), but the extent of inhibition of AOS parameters is greater (a decrease by 16%) [70]. Therefore, it is required to additionally adjust LPO and AOS parameters against the background of GCS use in BA patients, at the same time the ability of ILBI to normalize LPO processes is well enough confirmed [71].

The most significant AOS disorders are observed in hormone-dependent BA patients, but these patients show the best treatment outcomes after a course of ILBI-635 (633 nm wavelength, power of 3 mW, 20 min exposure time, 8–10 procedures per a treatment course daily). These results are correlated with a significant increase in the activity of antioxidant enzymes and a decrease in LPO intensity in RBC. Against the background of ILBI, a decrease in the average dosage of systemic GCS and a more relaxed transition to inhaled drugs have been observed. Patients who received ILBI against the background of traditional therapy as opposed to patients, who were traditionally treated, experienced faster clinical dynamics of the disease: decrease in the number of asthma attacks, replacement of full-scale attacks of asphyxiation with symptoms of dynamic bronchial obstruction, and decrease in cough intensity. Against the background of low-level laser therapy, there was a significant reduction in the need for bronchodilators and GCS doses. The average bed-occupancy rate was 10.7% less in such patients than in those who received only traditional therapy. The most pronounced improvement in respiratory function was observed in the group of patients with newly developed asthma [72–74].

Research by V.I. Korzhov et al. (1989) [75] showed that ILBI-635 in the comprehensive treatment of BA patients enables to achieve remission in 92.1% of cases and the phase of unstable remission in 7.9% of cases (in the control group 73.8 and 26.2%, respectively). At the same time, in patients of the main group (ILBI), the disappearance or decrease in the number of asthma attacks occurred in 5–6 days, while in the control group this result was achieved in 8–10 days. A reliable decrease (by 15.8%) was established in the concentration of molecules with average weight as compared to the initial level, and after treatment this parameter practically becomes equal to that in healthy donors – 238 ± 10.5 units. Analysis of the dynamics of accumulation of LPO products revealed an increase in their level in the studied patients; however, as a result of the treatment provided, the degree of normalization of both intermediate and final compounds is very significant. Thus, the content of lipid hydroperoxides decreased by 12.8% from the initial level, the decrease in the concentration of malondialdehyde (MDA) was even more significant - by 34%.

An increase in the conjugated diene concentrations is registered in all patients with moderate-severe asthma during exacerbation of the disease. These changes directly depend on asthma duration [73,74,76]. Comprehensive treatment, including ILBI procedures (633 nm wavelength, power of 2–3 mW, 20 min exposure time, 8–10 procedures per a treatment course daily), to a greater extent contributes to reducing the intensity of LPO processes as compared to traditional treatment.

The content of antioxidants in erythrocytes and plasma increases in the exacerbation phase in BA patients with short duration of the disease. In patients with the disease duration of more than 10 years against the background of an exacerbation, a decrease is registered in the concentration of antioxidants in the studied media, respectively. The application of ILBI-635 demonstrates a more pronounced tendency to normalize the studied parameters, i.e. this method is a powerful corrective tool for affecting patients' AOS as well [77–79]. Other authors also confirmed the best efficacy of laser therapy with disease duration of more than 10 years and a more severe course than with newly diagnosed BA [80].

All BA patients have changes in the cytokine profile, cellular and humoral immunity, the nature of which depends on the severity of the disease and on the available allergic reactions. With an increase in the severity of the disease, serum immunoglobulin (IgA) levels increase, while IgG and IgE decrease, which is accompanied by an increase in the number of granulocytes with the phenotype CD45 + CD66b + CD11b+ and enhanced phagocytic activity of neutrophils: the phagocytic number increases by 3–16 times, and the phagocytic index grows by 3.5–4 times as compared to healthy donors. Peripheral blood mononuclear cells isolated in the exacerbation phase of bronchial asthma are characterized by increased production of IL-4 (spontaneous production is 6.3 times as high, induced one is 4.8 times as high), IL-6 (spontaneous production is 2.4 times as high, induced one is 4.3 times as high) and IL-17 (spontaneous production increases by 23%, induced one - by 19%). The level of spontaneous production of IL-8 is reduced by 7%, while that of the induced one is increased by 7.5%. In this connection, recently, special importance has been attached to immunomodulatory therapy [81]. Low-level laser therapy has also fairly powerful capabilities in this component of the physiological regulation.

It has been shown that in patients with an infectious-dependent form of BA ILBI-635 contributes to the normalization of the number of E-rosetting cells, a decrease in the content of theophylline-resistant subpopulation of T-cells enhanced upon admission and an increase in the number of theophylline-sensitive subpopulation of T-cells, which leads to the normalization of theophylline-resistant-to-theophylline-sensitive T-cell ratio: E_th-r_-ROS/E_th-s-_ROS [82]. After a course of ILBI-635, dyspnea is reduced, the external respiratory function improves, a more rapid recovery of alveolar blood flow is observed, a distinct stimulating effect on the cell level parameters of phagocytic activity of neutrophils is reported [83,84].

It is most effective to administer ILBI-635 (633 nm wavelength, 1–2 mW, 30 min exposure time, 5–7 procedures per a treatment course daily) for patients with moderate depression of the T-cell immunity. ILBI can be successfully used as monotherapy in BA patients with mild disease and drug polyallergy. The distinct positive dynamics in the T-cell immunity naturally manifests itself in an accelerated and pronounced regression of the clinical implications of the disease [85].

The inclusion of ILBI -635 in the comprehensive therapy of infection-dependent BA increases the effectiveness of treatment: it accelerates the time for remission onset and increases its duration, reduces the frequency of exacerbations and enables to decrease the amount of drug therapy. Under the influence of ILBI, the inflammatory process subsides, which is reflected in ERF improvement (VC increases by 39.9%, FVC - by 27.9%, FEV1 - by 41.6%, MEF50 - by 42.0%, MEF75 - by 47.4%, MEF25 - by 58.3%), with marked fall in peripheral blood eosinophils and positive general clinical dynamics [86].

L.V. Vasilieva (1999) [87] showed the following effects of ILBI-635 in bronchial asthma:

– stimulation of β-adrenergic receptors;
– increase in the functional activity of lymphocytes and leukocytes, and phagocytic activity of neutrophils and monocytes;
– normalization of immunoglobulin levels and CIC;
– restoration of the aggregative state of blood.

The use of intravenous laser blood illumination in the comprehensive treatment of BA patients reliably improves bronchial patency compared with the results of conventional therapy. ILBI-635 has a corrective effect on the hemostatic system, mainly optimizes Hageman-kallikrein-dependent fibrinolysis, which determines an additional mechanism of its action. In addition, ILBI has anti-aggregation properties, reduces the coagulation potential, increases the antioxidant activity of the blood and decreases pre-beta cholesterol and beta cholesterol levels [88]. RBC morphometry and electrophoretic mobility measurement in BA patients after ILBI procedures show that the proportion of discocytes in the blood recovers almost to the norm [89].

Many experts are sure that the combination of plasmapheresis (PA) or enterosorption + laser blood illumination is one of the most effective options of therapy, including for BA patients [28,60,62,90–93].

Plasmapheresis combined with ILBI-635 allows for significant (by 60%) improvement of performance in patients with the most severe clinical course of asthma and concomitant autoimmune thyroiditis. Comprehensive therapy not only contributes to obtaining stable long-term remission, control over the BA symptoms, but also significantly reduces the antibody titer to the thyroid microsomal fraction [90].

Against the background of comprehensive therapy applying PA, UVBI and ILBI, in most cases, it is possible to achieve clinical remission in BA patients with a pronounced reduction in the amount of drug therapy, up to the discontinuation of hormonal drugs. It was shown that the therapeutic effect of the extracorporeal and laser therapy techniques is implemented by enhancing the therapeutic efficacy of sympathomimetic agents [94], and increasing the immunosorption and insulin-binding capacity of erythrocyte membranes [94,95].

Combining drug therapy of BA patients with PA and ILBI-635 it is possible to accelerate the onset of the disease remission through more rapid reverse development of asthma attacks, which results in the considerable increase in exercise tolerance and ERF normalization. With a significant reduction in the volume of drugs, the periods of remission are simultaneously extended. Combined treatment can prevent the development of complications by reducing the total doses of hormonal drugs or their discontinuation. LLLT contributes to more rapid stabilization of the bronchial receptor apparatus, in particular of β-adrenoreceptors, increasing their sensitivity to sympathomimetics and glucocorticoid drugs. Combination of ILBI and PA to treat patients with various forms of asthma corrects immune disorders and phagocytosis dysfunction, which is one of the main pathogenetic mechanisms causing a pronounced clinical effect and improving the course of the disease [60].

These outcomes are confirmed by other authors. Against the background of PA and ILBI-635, cough disappears in earlier periods and lung auscultation is normalized, while the dose of oral GCS is reduced, doubling the remission duration. After the course of LLLT, respiratory indicators that characterize the bronchial patency are normalized by the end of the 3rd week of treatment, providing rapid functional activation of the oxygen-dependent bactericidal system of blood neutrophils (NBT-test), which is associated with an additional increase in the T-lymphocyte suppressor potential, normalized immunoregulatory index and increased phagocytic activity of neutrophils [96–98].

According to A.S. Kuno (1994) [99], it is necessary to include immunomodulators in the treatment regimen in addition to PA and UVBI, which enable to prolong remission by stabilizing humoral immunity.

Along with ILBI, laser acupuncture is quite actively used to treat BA patients; more often, as we have noted above, as part of the comprehensive therapy with other methods of low-level laser therapy applied [52,93,100,101].

It was shown that the optimal time for local exposure in treating patients with allergic BA by pulsed IR LILI (890 nm wavelength, 5–7 W, 700–1600 Hz) is 60 s (paravertebrally), 300 s – supraclavicular region, by illuminating APs for 30 s (stimulation) and 60–90 s (suppression). The following acupuncture points were used: V10 (Tianzhu), V11 (Dazhu), V13 (Feishu), V12 (Fengmen), V15 (Xinshu), V17 (Geshu), CV22 (Tiantu), VC21 (Xuanji), VС20 (Huagai), VC17 (Shanchung).), VC16 (Zhongting), VC15 (Jiuwei), RP6 (Sanyinjiao), Е14 (Kufang), Е15 (Wuyi), Е36 (Zusanli), GI4 (Hegu), Р7 (Lieque), P11 (Shaoshang). Auricular points: AAP55 (Shen Men point, having general strengthening effect), AAP31 (Ping Chuan, Asthma point, relieving symptoms of asthma, calming panting, etc.), AAP13 (Adrenal Control point). Against the background of combined laser therapy, a positive ERF dynamics is observed, rhinocytogram indices are normalized, there is reduction in general and local eosinophilia), cutaneous and, especially, local sensitivity to specific allergens. The positive clinical effect of the therapy is accompanied by the immune normalization: T-lymphocyte and T-helper cell counts significantly increase, the level of serum immunoglobulins (IgA and IgG) raises, and the percentage of degranulated mast cells decreases [103].

Considerably worse results were obtained by M.A. Borodina (1999) [104], either because of a different acupuncture prescription, or because of the multifactorial effect on APs (simultaneous exposure to LILI, magnet and heat). An alternative prescription is given by foreign authors: P5 (Chize), Р7 (Lieque), P9 (Taiyuan), GI4 (Hegu), V13 (Feishu), V23 (Shenshu), Е36 (Zusanli), RP6 (Sanyinjiao), VG14 (Dazhui), VC17 (Shanchung). However, as is often the case, they use completely unacceptable LILI parameters [42], and perhaps for this reason their results are not the best ones.

I.E Yesaulenko et al. (2009) [105] recommended a simplified prescription to treat BA patients with concomitant chronic rhinosinusitis: GI4 (Hegu), Р7 (Lieque), GI20 (Yingxiang) and Р5 (Chize), ZP15 (Jiabi), VG23 (Shangxing), АAР22 symmetrically on alternate days, 10 procedures per a treatment course daily.

Laser acupuncture can significantly improve the results of outpatient treatment and rehabilitation of patients with light and moderate-severe asthma statistically significantly earlier than with conventional drug therapy.

Patients with severe BA demonstrate the best result with comprehensive therapy. The clinical course improves, bronchial sensitivity to sympathomimetics is restored, the need for β2-agonists, inhaled and systemic GCS is reduced, Short-Term Disability periods decrease by 5–7 days, remission duration is prolonged to 3 years, there is reduction in frequency of hospitalization by 1.3 times, emergency call incidence by 23%, and disability retirement rate to 12%.

The restoration of impaired histochematic barrier and systemic changes in the synthesis and utilization of biogenic amines by the blood cells that stimulate the production of mature heparin, which binds inflammatory mediators – histamine and serotonin, as well as an excess of catecholamines is the basic mechanism of therapeutic action of LILI. Heparin has anti-inflammatory value and removes the blockade of β2-adrenergic receptors, and catecholamines produce bronchodilation, by binding to β2-adrenergic receptors [51].

Summing up some intermediate result of the brief review, we have systematized the results of various studies (Table 2), which clearly demonstrate the normalizing effect of laser illumination on virtually all known pathogenetic mechanisms of asthma development, therefore, there is no doubt that laser therapy can and should be used as the basic method of therapy. However, there is still a very important issue about the optimization of laser therapy parameters; the answer to this question helps gain knowledge of the mechanism of therapeutic action of LILI and some rules. Sometimes it is hindered by incorrect description of materials and research methods [16], which raises doubts about the reliability of their results, but there are so many publications (Table 2 gives only a small part of them as an example) that it is easy to draw conclusions about the optimal techniques.

Laser blood illumination, as we said above, is the most commonly used method for treating BA. While the issue with the optimal power for the “classical” ILBI-635 technique (633 nm wavelength) can be considered solved (it should be 1–3 mW), the issue with the exposure time is more complicated. Previously, laser illumination was performed by many authors for 30 min or even more [17,61,85], although it has long been unequivocally proven that the exposure time for this mode of ILBI should not exceed 15–20 min. Moreover, this conclusion was made not only for bronchial asthma [76], but also for other pathological processes and diseases [20].

It is known that in order to get the best treatment outcome it is necessary to combine various methods of LLLT for topical and systemic action [20], but this is especially important if the patient has several diseases.

Comparative evaluation of the clinical efficacy of laser therapy in the comprehensive treatment of BA patients against the background of hormonal disorders confirmed that precisely the combined methods are most optimal when using laser illumination techniques of both primarily topical and systemic action on the entire body as a whole [110; 122; 123]. Combining ILBI-635 and external LILI for patients with various hormonal disorders characteristic of asthma improves the quality of patients' life [5]. Long-term treatment of BA patients using systemic GCS increases the risk of developing osteoporosis. Low-level laser therapy (ILBI-635) is an effective means of preventing complications and helps normalize testosterone and estradiol levels [124]. Combining ILBI-635 and external LILI is most effective for osteoporosis prevention in patients taking GCS [44].

The availability of BA determines the peculiarities of hypertension progression, since a sharp rise in blood pressure often occurs at the time of a suffocation attack or an increase in bronchial obstruction, leading to negative consequences. Laser therapy in this case acts as a non-specific therapeutic factor, providing a pronounced double effect, promoting the improvement of the bronchial patency of the large, medium and small bronchi due to a pronounced anti-inflammatory, bronchodilatory, anti-edematous, antioxidant action, simultaneously normalizing blood pressure and preventing its sharp jumps [117,119,120].

The efficacy of combined LLLT for patients with asthma and stage 1 hypertensive disease is 80%, and for patients with asthma and stage 2 hypertensive disease is 70%, respectively. It is recommended to expose consistently the projection of the vasomotor center of the brain – the region of the posterior cranial fossa (1 min, 800–1500 Hz frequency), the projection of kidneys (for 5 min per each, 80–1500 Hz frequency), the projection of the lower lung lobes symmetrically (2 min, 80 Hz frequency) with pulsed IR LILI (904 nm wavelength, 100 ns light pulse duration, 8–10 W) paravertebrally at the C_4_–C_6_ level, at the Th_2_–Th_6_ level (for 1 min per each, 80–150 Hz), 10 procedures per a treatment course daily [117].

A special situation develops in BA patients with hormonal disorders and metabolic syndrome (MS). In case of bronchial disorders accompanied by bronchial obstruction, the leading role in the genesis of the impaired functional status of the adrenal glands is assigned to chronic hypoxia and hypoxemia (as a result of impaired bronchial patency and alveolar hypoventilation), which are the trigger mechanisms of stress. Rapid response activation of the hypothalamic-pituitary-adrenal system causes a standard non-specific reaction in the form of adrenocortical hypertrophy, lymph node atrophy, etc. In the conditions of pathology, the adaptive role of a number of hormones consists primarily in their influence on the development of inflammatory processes. The possibility of correcting the neuroendocrine system work after laser therapy is confirmed by the normalized serum aldosterone levels in such patients [6].

There are common key links in the pathogenesis of BA and MS: energy dependence of these processes, increased consumption of plastic material with involvement of the immune system in pathological reactions, activation of cytokine mechanisms, intense work of the endocrine system with active release of hormones and neurotransmitters into the blood. With this in mind, it can be assumed that a proper correction of MS contributes to the development of positive dynamics for the bronchial asthma progression. Laser therapy methods allow obtaining good treatment outcomes and are an effective means of preventing the development of complications [113–115]. The developed method of combined therapy for BA patients can increase the treatment efficacy and shorten the treatment duration, prevent side effects, correct hormone levels and reduce the dose of drugs used. Against the background of drug therapy, patients are exposed to NLBI and pulsed IR LILI in the projection of the adrenal glands. In this case, the NLBI is performed for 15 min at a wavelength of 635 nm. Before the 1st and 5th procedures of exposure to the pulsed IR LILI, the levels of cortisol and estradiol in women and testosterone in men are determined. At cortisol levels below 230 nmol/l and testosterone levels below 500 ng/dl in men and estradiol levels below 30 pg/ml in non-menopausal women and below 15 pg/ml in menopausal women, a pulse repetition frequency of 150 Hz is used. At the cortisol levels ranging from 230 to 750 nmol/l and estradiol levels ranging from 30 pg/ml to 160 pg/ml in women and testosterone ranging from 260 to 1593 ng/dl in men, a pulse repetition frequency of 80 Hz is used. The total time of the procedure is no more than 18 min. The treatment course consists of 10–12 daily procedures [125].

External illumination by pulsed IR LILI is carried out in BA patients either in the projection of the upper respiratory tract to stimulate β-adrenergic receptors, eliminate inflammation and bronchospasm, or in the projection of the adrenal cortex to stimulate the release of steroid hormones [26,109,110,114]. Most often, both variants are used with the aim of simultaneously affecting several mechanisms of the disease pathogenesis.

The possibility of using reflex zones in treating patients with allergic asthma for stimulation of blood circulation and trophism is noted. In this case laser illumination is performed paravertebrally in the C_7_–Th_6_ area in addition to exposing the projection of the inflammatory infiltrate and the adrenal cortex. When using this technique, persistent remission (no recurrence within 2–3 years) was observed in 14% of patients (with mild progression of the disease), who simultaneously stopped taking GCS. Relative remission, characterized by the absence of full-scale asphyxiation attacks during 1.5–2 years and occasional difficulties in breathing, occurred in 65% of patients [26].

We have shown the need to control the hormone content in the process of lasertherapy of BA patients. A method of exposure was proposed, consisting in illuminating two paravertebral and intercostal regions and two Krenig fields by pulsed IR LILI (890 nm wavelength, 8–10 W/cm^2^ power density, 1500–3000 Hz frequency, 5 min exposure time, 10 procedures per a treatment course daily). This technique is characterized by the use of additional modulation in the well-known “BIO” mode (synchronization of changes in the LILI power with patient's pulse and respiration frequency). In addition, after the 1st and 3rd LLLT procedures, the content of 11-oxicorticosteroids (11-OCS) is determined; in case of a decrease in their content further treatment continues. In the majority of patients (80%) with a good and satisfactory effect of LLLT, there was a significant decrease in the level of 11-OCS in the blood plasma and daily urine after the 1st procedure, and this trend continued until the end of the treatment. In patients with an unsatisfactory treatment outcome, the level of 11-ACS either did not change or tended to increase. The mode of illumination (power, frequency) was modified in a number of patients based on the change in the level of 11-OCS after the first procedure. In some cases, the dynamics of 11-OCS in the absence of a pronounced clinical effect suggested the indirect effect of LILI on metabolic processes in the lungs, which was confirmed later on by significantly better curability of patients when using drug therapy [126].

When treating BA patients with metabolic syndrome, Kryuchkova et al. (2011) [127] recommended combining ILBI with external exposure to green light. Although in our opinion, this is hardly more effective than pulsed infrared LILI, even if we consider the situation only in terms of the depth of penetration, not to mention the special “healing properties” of coherence [128]. Light in the green region of the spectrum, regardless of the mono-chromaticity degree, is almost completely absorbed already in the upper layers of the skin, penetrating no more than a few millimeters, and therefore, it cannot directly affect the bronchi [20]. Although the issues of implementing the reflex mechanism and psychotherapeutic effects may well be discussed.

Endobronchial technique, which was used by some experts [129,130], did not find practical application due to the complexity of implementation with the worst treatment outcomes, which are achieved by using other methods of laser illumination.

Summing up the literature review, knowing the disorders that underlie the pathogenesis of the disease (Fig. 1), as well as the biomodulating mechanisms of the LLLT, we have drawn a scheme (Fig. 2) that clearly illustrates the basic mechanisms underlying the treatment of patients with bronchial asthma.

It seems promising to use low-intensity illumination of the extremely high-frequency range and laser light (EHF-laser therapy) [131], some progress has already been achieved in this direction [132,133], but the optimization of exposure parameters is clearly required.

Proceeding from the above data and an understanding of the methodology as a whole, we present laser therapy techniques with optimal parameters that are recommended for the treatment of patients with various forms of bronchial asthma, once again emphasizing the need for combination and variation.

**ILBI-635 Technique:** Matrix and Lasmik laser therapeutic devices, KL-ILBI-635-2 laser emitting head (635 nm wavelength, light guide output power of 1.5–2.5 mW, 15–20 min exposure time), 5–10 procedures per a treatment course daily [83,87]. Based on the well-known fact that the LLLT efficacy is observed with a disease duration exceeding 10 years and a more severe course than with newly diagnosed BA, it is recommended to reduce the exposure time for this category of patients down to 3–5 min for ILBI-635 [80].

It is also required to reduce the exposure time down to at least 7–10 min in all children (5–7 procedures per a treatment course daily) [106]. Although, in our opinion, in pediatrics, it is preferable to use a non-invasive option of the laser blood illumination technique – in the projection of the supraclavicular region on the left (the technique parameters depend on age).

For the last 8–10 years such “classic” version of ILBI has been actively supplanted by a more effective combined technique, which allows both influencing the immune system and activating metabolism. It is highly recommended to combine laser blood illumination with plasmapheresis.

**ILBI-635 + LUVBI**^®^ **Technique:** Matrix and Lasmik laser therapeutic devices, KL-ILBI-635-2 laser emitting head (red spectrum, 635 nm wavelength, light guide output power of 1.5–2 mW, 10–20 min exposure time) and KL-ILBI-365-2 laser emitting head 365–405 nm wavelength, light guide output power of 1.5–2 mW, 3–5 min exposure time). It is recommended to perform 10–12 procedures per a treatment course daily with mode alternation in a day [20].

**ILBI-525 + LUVBI**^®^ **Technique:** Matrix and Lasmik laser therapeutic devices, KL-ILBI-525-2 laser emitting head (green spectrum, 525 nm wavelength, light guide output power of 1.5–2 mW, 7–10 min exposure time) and KL-ILBI-365-2 laser emitting head 365–405 nm wavelength, light guide output power of 1.5–2 mW, 3–5 min exposure time). It is recommended to perform 10–12 procedures per a treatment course daily with mode alternation in a day [20]. The most advanced version of ILBI, which has proven its effectiveness in many diseases; however, it is necessary to conduct appropriate clinical studies and test the applicability in bronchial asthma.

According to S.V. Papkov (2002) [80], regardless of the LLLT technique used, in the overwhelming majority of cases, the course treatment of BA patients should be limited to 8 procedures daily for 10–15 min, since longer exposure has a stressful effect, leading to worsening of some autonomic parameters of the body. From this viewpoint, ILBI-525 + LUVBI^®^ technique is also preferable to ILBI-635.

**Non-invasive laser blood illumination (NLBI):** *The technique is not applied on the same day as ILBI.* Wavelength of 635 nm, pulsed mode, ML-635-40 matrix laser emitting head (Matrix or Lasmik device, eight 4–5 W laser diodes), power of 50–80 W, power density of 4–5 W/cm^2^, frequency of 80 Hz (variation is not allowed).

**Laser acupuncture:** Wavelength of 635 nm, continuous or modulated operation mode, and the output power at a special acupuncture nozzle is 2–3 mW, to be exposed for 20–40 s on one corporal point, the prescription is selected individually or on the recommendation of specialists.

**In the projection of the internal organs** Wavelength of 904 nm, pulsed mode, ML-904-80 matrix laser emitting head (Matrix or Lasmik device, eight 10 W laser diodes), power of 50–80 W, power density of 8–10 W/cm^2^, frequency of 80 Hz (variation is allowed).

Localization (projection) and exposure time:

– upper respiratory tract - 2 or 5 min, 1–2 areas**;**
– thymus - 1 min;
– adrenal cortex - 2 or 5 min per area symmetrically.

**Paravertebrally:** Wavelength of 904 nm, pulsed mode, LO-904-20 matrix laser emitting head with a mirror expender (Matrix or Lasmik device, one laser diode), power of 15–20 W, power density of 10–15 W/cm^2^, frequency of 80 Hz (variation is not allowed) at the level of C_4_–C_6_ and Th_2_–Th_6_ symmetrically, 1 min per each region.

**Endonasal technique:** The application of the technique is associated with the execution of certain mandatory rules. Continuous LILI of the red spectrum (635 nm wavelength, light guide output power of 3–5 mW) through the light guide or directly via the laser emitting head to expose for 2 min per one nasal passage. The efficacy and validity of the technique is beyond doubt [64; 134]; however, it is necessary to carefully consider its purpose, to control the technique parameters, especially the exposure time.

In case of bronchial asthma with concomitant chronic rhinosinusitis, another technique is used, although the nasal area is exposed. Firstly, only *pulsed IR* LILI (890–904 nm, 5–10 W, 80 Hz) should be used, secondly, there is other localization: projections of maxillary sinuses and/or frontal sinuses and/or ethmoidal sinuses (depending on the localization of the inflammatory process according to x-ray or computed tomography data) on both sides, for 2 min each [121].

Most often, 10–15 daily procedures are recommended per a treatment course. Although there are other options, both upwards and downwards, we agree precisely with this approach. Fewer procedures will not allow obtaining a stable clinical effect, and more does not make sense according to the logic of the known patterns of chronobiology and chronomedicine.

The issue of alternating different techniques is also very important. In our opinion, if guided by the well-known rule of limiting the total procedure time to 20–25 min, then *on one day 2-3-4 different options of laser illumination* may and should be performed. The opinion of some experts that it is optimal to combine ILBI-635 with the exposure to pulsed IR LILI in the projection of the trachea region (supra-dorsal) and paravertebral every other day, 10–12 procedures per a treatment course daily [122], is most likely related to the wrong exposure time selected for ILBI.

Thus, low level laser therapy for BA patients with proper prescription and appropriate procedures, results in sustained remission throughout the patient's entire life. Moreover, drugs are not excluded from the treatment regimen, but are considered as part of the auxiliary treatment to provide emergency care in case of unforeseen and/or provoked exacerbation. Courses of prophylactic laser therapy are recommended: 3–5 procedures daily or every other day, at least 3–4 times a year [26,36,60,82,85,86]. It is preferable to use two techniques, most often ILBI and topical exposure. A course of prophylactic treatment is additionally prescribed for patients with allergic BA before the onset of seasonal exacerbation.

## Figures and Tables

**Fig. 1:**
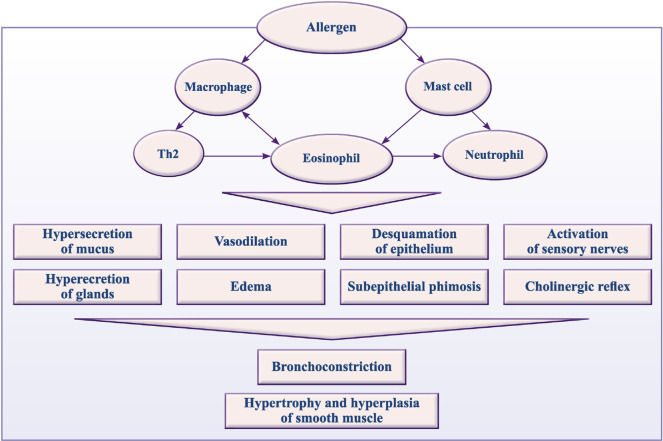
Inflammation plays the central role in BA pathogenesis.

**Fig. 2:**
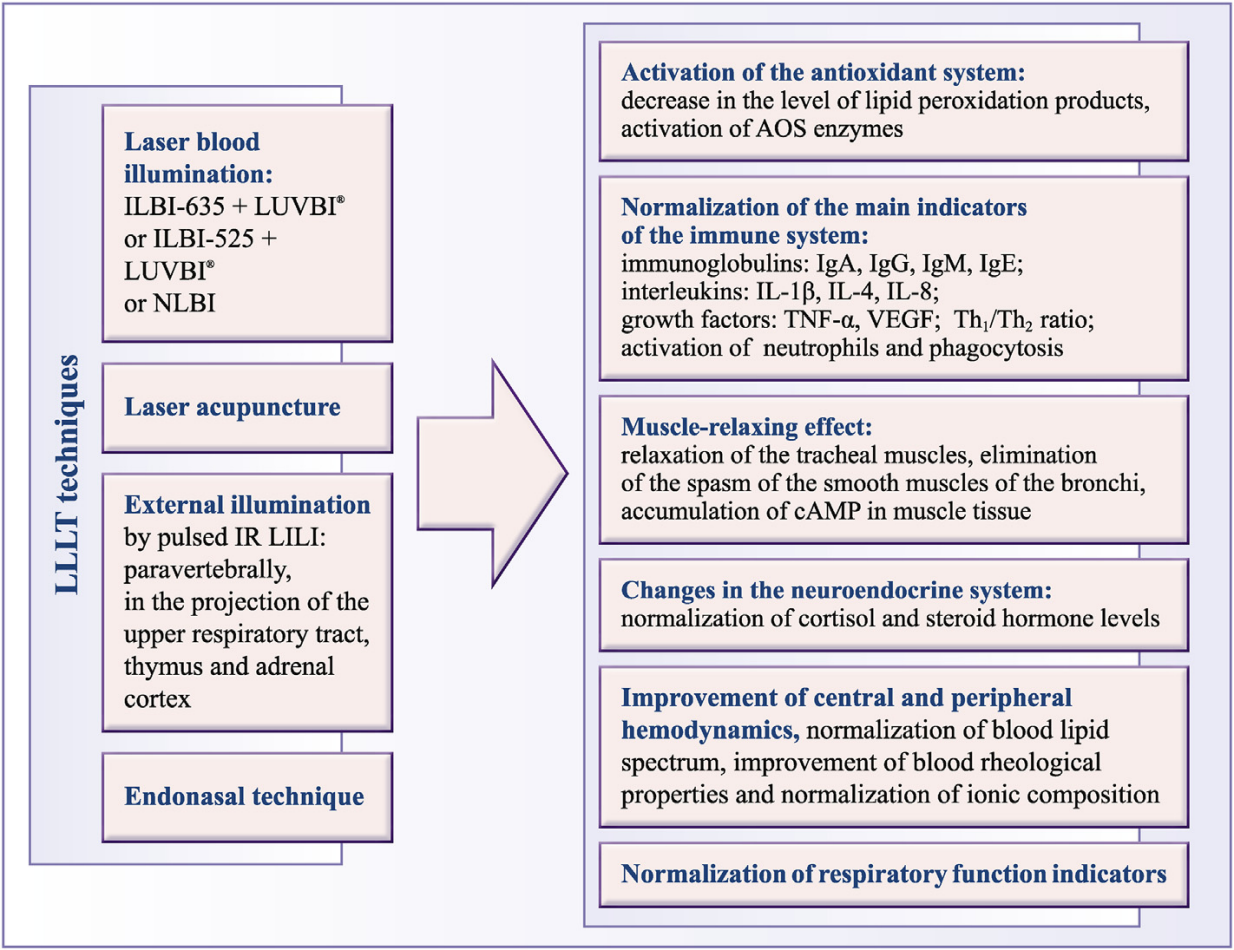
Low-level laser therapy mechanisms in the treatment of BA patients.

**Table 1:** Dynamics of reducing the frequency of asthma attacks (AA) and the need for β2-agonists in patients with asthma depending on the severity of the disease and laser therapy techniques [67].

Methods of treatment/indicators	Light		Medium		Strong	
	AA	β2-agonists	AA	β2-agonists	AA	β2-agonists
Group 1	4,2	2,3	2,5	2,5	–	–
Group 2	3,6	3,5	4,2	1,7	1,5	2,5
Group 3	–	–	2,0	1,4	2,5	2,9

**Table 2:** Pathogenetic justification of the effectiveness of LLLT in patients with BA.

Form of BA; clinical outcome	Indicator	LLLT technique (number of daily procedures)	Reference
**Antioxidant System**			
ABA, children; reduction of bronchospasm attacks and dyspnea, up to the complete disappearance	Activation of AOS, reduction of the level of primary and final LPO products	ILBI-635 (3–5)	[106]
MBA; earlier normalization of the main clinical and laboratory signs, reduction of the dose of GCS	Reduction of MDA, lipid hydroperoxides, increase in SOD	ILBI-635, external pulsed IR LILI: paravertebrally Th_3_–Th_5_, at the II and III intercostal space, symmetrically, projection of the adrenal glands (14)	[87]
IABA; a significant decrease in the amount of medications taken and a reduction of the number of asthma attacks	Normalization of the work of AOS for all studied parameters (DC, MDA, Schiff bases, SOD, catalase, glutathione peroxidase, glutathione reductase)	Enterosorption and ILBI -635 (10)	[62]
ABA, IABA, MBA; reduction of doses of hormonal drugs and cancellation, reduction of the days of disability, lengthening the terms of remission 2.4 times	Decrease in the content of LPO products (DC, MDA), increase in enzyme activity (SOD, catalase), stabilization of cell membranes	ILBI-635 (5–12)	[66]
*In vivo*, mice	Reduction of ROS content, decrease in activity of NO-synthase in bronchoalveolar lavage fluid, increase in catalase activity, SOD, glutathione peroxidase, NADPH oxidase, and Nrf2 transcription factor	660 nm, 30 mW, 5 min (1)	[55]
*In vitro*, U937 cells	Suppression of glucocorticoid resistance induced by oxidative stress, inhibition of TNF-α and IL-8 secretion through an increase in cAMP and inhibition of the PI3K signaling pathway	660 nm, 17,85 mW/cm^2^, 60 s (1)	[58]
**Immune system**			
Not indicated	Decrease in eosinophil count	Laser acupuncture (10–20)	[107]
ABA, children; reduction of bronchospasm attacks and dyspnea, up to the complete disappearance	Normalization of the ratio of T-and B-lymphocytes	ILBI-635 (3–5)	[106]
IABA; decrease in skin and, especially, local sensitivity to specific allergens	Reduction of general and local eosinophilia is accompanied by normalization of immunity: the content of T-lymphocytes, T-helpers increases, the level of serum immunoglobulins A and G increases, the percentage of degranulated mast cells decreases	Laser acupuncture (10–15)	[103]
ABA	Normalization of *IgA, IgG* and *IgM* levels	LBI, laser acupuncture, in the projection (10)	[18]
MBA; a significant decrease in the amount of medications taken and a reduction of the number of asthma attacks after LLLT course	Normalization I*gA, IgG, IgM, IgE* levels, activation of phagocytosis	PA and ILBI-635 (4–7)	[60]
ABA, IABA with concomitant autoimmune thyroiditis; increase in the duration of remission	Reduction of antibody titer to the microsomal fraction of the thyroid gland	PA and ILBI-635 (3–5)	[90]
*In vitro*	Changes in the morphofunctional state of lymphocyte membranes	633 nm (1)	[108]
ABA, MBA; decrease in the frequency of attacks	Decrease in eosinophil count	Endonasal, topical, ILBI (10)	[63,64]
ABA, children	Reduction of *IgE* level	Laser acupuncture (10)	[52]
MBA and COB; rapid regression of clinical symptoms	Activation of T-cellular component of immune system	ILBI-635 (5–7)	[85]
MBA, children; clinical symptoms are reduced 3–6 days faster	Reduction of IgE, VEGF, IL8, IL4 levels, CD4+ lymphocytes, increase in CD8+ lymphocytes	Laser acupuncture (10)	[32]
IABA	Normalization of the ratio of Eth-r-ROS/Eth-s-ROS	ILBI-635 (5–8 on alternate days)	[82]
IABA; remission occurs one week faster, its duration increases; the severity of the disease decreases and the frequency of exacerbations is reduced 2 times; the dose of oral corticosteroids is reduced	Activation of neutrophils of peripheral blood	PA and ILBI-635 (8–10)	[96]
Not indicated	Reduction of IgG, CIC levels, increase in phagocytosis and normalization of the immunoregulatory index of the T-system of immunity	PA and ILBI-635 (3–4)	[98]
ABA, children; absence of severe asthma attacks and the frequency of attacks of moderate and mild severity are 1.8–2.5 times less	Normalization of levels of immunoglobulins of the main classes and reduction of the initially high level of IgE, normalization of phagocytosis and levels of proinflammatory cytokines in blood serum	External pulsed IR LILI (10)	[36]
ABA, adults and children	Increase in metabolic and mitotic activity of lymphocytes, neutrophil phagocytosis, changes in the expression and affinity of E-receptors of lymphocytes, a decrease in IgM concentration in blood serum	External pulsed IR LILI (5–8)	[29]
ABA, children; decrease in the number of asthma attacks 4 –6 times, reduction of the severity of the disease	Normalization of *IgA, IgG, IgM, IgE, IL-1β* and *TNF*-α levels	External pulsed IR (890 mm) LILI (10)	[38]
ABA, HBA	Decrease in the number of eosinophils, normalization of IgA, IgG and IgM levels	Laser acupuncture (10–15)	[51]
Not indicated	Normalization of T-cell immunity	ILBI-635 (5)	[69]
ABA, IABA, MBA; normalization of sensitivity and reactivity of the bronchi	Normalization of T-lymphocyte differentiation, increase in T-suppressor activity, decrease in IgE production	ILBI-635 (5–12)	[66]
Illumination of the blood of patients with MBA *in vitro*	Increase in phagocytic index and neutrophil count	633 nm, 20 mW	[13]
Not indicated, children; the number of exacerbations decreased 3 times, the need for antibiotics decreased 3.7 times	Normalization of almost all investigated parameters of the immune status (CD3+, CD4+, CD8+, CD16+, CD20 +,IgA, IgG, IgM)	Externally on several areas by continuous LILI of red spectrum (633 nm) and pulsed IR (890 nm) LILI (7-10)	[41]
*In vivo*, mice	Reduction of *IgE* level	660 nm, 30 mW, 5 min (1)	[55]
*In vivo*, mice	Decrease in the number of eosinophils and bronchial hyperactivity through the expression of the *RhoA* gene, reduction of allergic lung inflammation through the expression of the *STAT6* gene	660 nm, 30 mW, 5 min (1)	[57]
*In vivo*, rats	Decrease in the number of eosinophils, *IL-4* and *IgE* levels, increase in the production of IFN-γ, the ratio of T-helpers *Th_1_/Th_2_* is normalized	810 nm, 20 mW/cm^2^, 20 min (21)	[59]
**Muscle tone**			
*In vivo*, rats	Relaxation of the inflammatory smooth muscle of the trachea, TNF-α inhibition, cAMP accumulation	650 nm, 31,25 mW/cm^2^, 42 and 300 s (1)	[54]
*In vivo*, rats	Decrease in cholinergic hyperactivity, elimination of bronchial smooth muscle spasm, reduction of the expression of mRNA TNF-α	655 nm, 31,25 mW/cm^2^, 42 s and 5 min (1)	[56]
**Neuroendocrine system**			
ABA	Initial low blood cortisol increases	LBI, laser acupuncture, in the projection (10)	[18]
MBA; a significant decrease in the amount of medications taken and a reduction of the number of asthma attacks after LLLT course	The sensitivity of β-adrenoreceptors to sympathomimetics and glucocorticoid drugs is increased	PA and ILBI-635 (4–7)	[60]
MBA	Increase in testosterone levels in men and estradiol in women	Pulsed IR LILI in the projection of the adrenal glands and NLBI (10)	[4,5]
Aspirin BA; 2 times lower doses of GCS and 2–2.5 times of β2-agonists	Increase in ACTH and cortisol levels in the blood	Hemosorption and ILBI-635 (8)	[91]
Not indicated; reduction of GCS doses	Normalization of aldosterone levels, increase in steroid hormones levels in the blood	Pulsed IR in the projection of the upper respiratory tract (10–15)	[6,109,110]
ABA, HBA	Initially reduced levels of cortisol and catecholamines in the blood increase, initially increased levels of histamine and serotonin are reduced	Laser acupuncture (10–15)	[51]
Not indicated	Increase in mineralcorticoid function of the adrenal cortex, normalization of the ionic composition of blood (potassium, sodium)	Laser acupuncture (15–20)	[111]
Not indicated	Increase in levels of 11-oxycorticosteroids (11-OCS) in the blood	ILBI-635 (5)	[69]
**Vascular system, hemorheology**			
ABA, children; reduction of bronchospasm attacks and dyspnea, up to the complete disappearance	Improvement of the structure of erythrocyte membranes	ILBI-635 (3–5)	[106]
Not indicated	Lengthening the blood clotting time, reduction of fibrinogen concentration and increase in fibrinolytic activity of blood	Laser acupuncture (10–20)	[112]
Not indicated; a significant decrease in the amount of medications taken and a reduction of the number of asthma attacks after LLLT course	Improvement of the rheological properties of blood, increase in the deformability of erythrocyte membranes, decrease in the content of echinocytes	ILBI-635 (10)	[61]
ABA	Changes of indicators of central hemodynamics	LBI, laser acupuncture, in the projection (10)	[18]
MBA, children	Normalization of erythrocyte and platelet parameters, restoration of endothelium-dependent characteristics (endothelin-1 and circulating endotheliocytes)	Pulsed IR LILI in the projection of lungs (10)	[30,31]
MBA + hypertensive disease	Normalization of blood lipid spectrum	ILBI-635 (−)	[77]
IABA; remission occurs one week faster, its duration increases; the severity of the disease decreases and the frequency of exacerbations is reduced 2 times; the dose of oral GCS is reduced	Improvement of central and peripheral hemodynamics	PA and ILBI-635 (8–10)	[96]
Not indicated, combination with MS	Normalization of blood lipid spectrum	ILBI-635 (8–10)	[113–115]
IABA; improvement of bronchial patency	Hageman-kallikrein-dependent fibrinolysis is optimized, anti-aggregation effect appears, coagulation potential decreases, blood antioxidant activity increases, pre-beta cholesterol and beta cholesterol levels decrease	ILBI-635 (5)	[88]
Not indicated	Normalization of blood lipid spectrum	Laser acupuncture (10–20)	[116]
IABA	Restoration of the form of erythrocytes, an increase in the proportion of discocytes in the blood	ILBI-635 (10)	[89]
HBA, IABA; reduction in the amount of drug therapy up to cancellation of hormonal drugs	Increase in immunosorption and insulin binding ability of erythrocyte membranes	PA, UVBI and ILBI-635 (10)	[94]
ABA, IABA; broncholytic effect, cancellation of prolonged β2-agonists and reduction of doses of systemic GCS	Favorable effect on central hemodynamics, microcirculation and rheological properties of blood	ILBI-635 (−)	[67]
IABA; the main symptoms of the disease are stopped more quickly with an earlier cancellation or reduction of the dose of drugs	LLLT contributes to a more complete recovery of foregrams, a rapid decrease in elevated levels of sialic acids, seromucoids, ceruloplasmin, and the activity of the kinin-kallikrein system	Laser acupuncture, continuous LILI (633 nm) on reflex zones (10–19 depending on the severity)	[26]
**Respiratory function (normalization of indicators)**			
IABA	VC, FVC	Laser acupuncture (10–15)	[103]
ABA	VC, FVC, MEF_25-75_, FEV_1_, FEV_1_/FVC	LBI, laser acupuncture, in the projection (10)	[18]
MBA; earlier normalization of the main clinical and laboratory signs, reduction of the dose of glucocorticoids taken	VC, FVC, MEF_25-75_, FEV_1_, FEV_1_/FVC	ILBI-635, external pulsed IR LILI: paravertebrally Th3–Th5, at the II and III intercostal space symmetrically, the projection of the adrenal glands (14)	[87]
MBA	FVC, FEV_1_, FEV_1_/FVC	Pulsed IR LILI in the projection of the adrenal glands and NLBI (10)	[4]
ABA, children	PEF, FEV_1_	Laser acupuncture (10)	[52]
ABA; elimination of bronchospasm	VC, FVC, FEV_1_, FEV_1_/FVC, PEF, etc.	Laser acupuncture (10–20)	[100]
MBA, children	FVC, PEF	Pulsed IR LILI in the projection of lungs (10)	[30,31]
ABA, MBA; decrease in the frequency of attacks	VC, FVC, MEF_25-75_, FEV_1_, FEV_1_/FVC, PEF	Endonasal, topical, ILBI (10)	[63]
MBA and COB; rapid regression of clinical symptoms	VC, FVC, MEF_25-75_, FEV_1_, FEV_1_/FVC	ILBI-635 (5–7)	[85]
MBA, children; clinical symptoms are reduced 3–6 days faster	VC, FVC, MEF_25-75_, FEV_1_, FEV_1_/FVC	Laser acupuncture (10)	[32]
ABA, children; absence of severe asthma attacks and the frequency of attacks of moderate and mild severity are 1.8–2.5 times less	VC, FVC, MEF_25-75_, FEV_1_, FEV_1_/FVC, PEF	External pulsed IR LILI (10)	[36]
BA and hypertensive disease	MEF_75_, FEV_1_, PEF_25-75_, normalization of blood pressure	External, NLBI (10)	[117]
MBA	FEV_1_, PEF, reduction of endogenous intoxication	NLBI, laser acupuncture (12–14)	[118]
ABA, IABA, MBA	VC, FVC, MEF_25-75_, FEV_1_, FEV_1_/FVC	NLBI, ILBI-635 (8)	[80]
ABA, IABA, MBA; elimination of bronchial obstruction syndrome	VC, FVC, FEV_1_, FEV_1_/FVC, PEF	ILBI-635 (5–12)	[66]
ABA, HBA; the need for β2-agonists, inhalation and systemic GCS decreases, the period of temporary disability decreases by 5–7 days, the duration of remission increases up to 3 years	VC, FVC, FEV_1,_ MEF_50,75_	Laser acupuncture (10–15)	[50,51]
ABA, children; absence of severe asthma attacks and reduction of the frequency of attacks of moderate and mild severity	VC, FVC, MEF_25-75_, FEV_1_, FEV_1_/FVC, PEF	External pulsed IR LILI (10)	[40]
IABA	VC, FVC, MEF_25-75_, FEV_1_, FEV_1_/FVC, PEF	ILBI-635 (5)	[86]
MBA; improvement of bronchial patency of large, medium and small bronchi due to a pronounced anti-inflammatory, bronchodilator, anti-edematous, antioxidant action	VC, FVC, MEF_25-75_, FEV_1_, FEV_1_/FVC, PEF	NLBI (10)	[119,120]
MBA with rhinosinusitis	VC, FVC, MEF_25-75_, FEV_1_, FEV_1_/FVC, PEF	Pulsed IR LILI endonasally	[121]
Not indicated, children; improvement according to GINA criteria in 91.7% of patients, reduction of doses of medications	VC, FEV_1_, FEV_1_/FVC	Laser acupuncture (10)	[42]
Not indicated, children	VC, FEV_1_, FEV_1_/FVC, PEF	Laser acupuncture (10)	[43]
Not indicated, children	FEV_1_, MEF_25_	Laser acupuncture (10)	[44]
Not indicated, children	PEF	Laser acupuncture (10)	[46]
Not indicated, children	VC, FVC, MEF25-75, FEV_1_	Laser acupuncture (10)	[48]
Not indicated, children; improvement of the quality of life	PEF, FEV_1_	Laser acupuncture (10)	[49]

Notes: ABA – atopic BA; BA – bronchial asthma; COB – chronic obstructive bronchitis; ILBI - intravenous laser blood illumination; HBA – hormone dependent BA; GCS – glucocorticosteroids; DC – diene conjugates; VC – vital capacity; IABA – infectious-allergic BA; IFN – interferon; LLLT - low-level laser therapy; MDA – malondialdehyde; MEF_25-75_ – maximal expiratory flow at 25%–75%; MS – metabolic syndrome; NLBI- non-invasive (percutaneous) laser blood illumination; FEV_1_ – forced expiratory volume in 1 s; FEV_1_/FVC – the Tiffeneau index; PA – plasmapheresis; LPO – lipid peroxidation; PEF – peak expiratory flow rate; MBA – mixed BA; SOD – superoxide dismutase; FVC – forced vital capacity; cAMP – cyclic adenosine monophosphate; CIC – circulating immune complexes; GINA – Global Initiative for Asthma; *IL* – interleukin; *NF-κB* – nuclear factor-kappa *B*; *NO* – nitrogen oxide; *PI3K* – phosphoinositide 3-kinase; *TNF*-α – tumor necrosis factor-alpha; *VEGF* – vascular endothelial growth factor.
